# The Gaps Between the Self and Professional Evaluation in Mental Health Assessment of COVID-19 Cluster Cases

**DOI:** 10.3389/fpsyg.2021.614193

**Published:** 2021-08-31

**Authors:** Ligai Liu, Xin Wang, Yiwei Hao, Jingming Yang, Di Yang, Xuefei Duan, Gaoli Fang, Bing Han, Chunguo Jiang, Junnan Li, Yao Liu, Yang Wang, Rui Song

**Affiliations:** ^1^Liver Disease Center, Beijing Ditan Hospital, Capital Medical University, Beijing, China; ^2^Beijing HuiLongGuan Hospital, Peking University, Beijing, China; ^3^Department of Medical Records and Statistics, Beijing Ditan Hospital, Capital Medical University, Beijing, China; ^4^Department of Infectious Diseases, Beijing Ditan Hospital, Capital Medical University, Beijing, China; ^5^Department of General Medicine, Beijing Ditan Hospital, Capital Medical University, Beijing, China; ^6^Departments of Otolaryngology, Beijing Ditan Hospital, Capital Medical University, Beijing, China; ^7^Department of Respiratory and Critical Care Medicine, Beijing Chaoyang Hospital, Capital Medical University, Beijing, China; ^8^Statistics Room, Beijing Ditan Hospital, Capital Medical University, Beijing, China; ^9^Department of Integrative Traditional Chinese Medicine and Western Medicine, Beijing Ditan Hospital, Capital Medical University, Beijing, China; ^10^Department of General Surgery, Beijing Ditan Hospital, Capital Medical University, Beijing, China

**Keywords:** SARS-CoV-2, COVID-19, self-rating scale, professional evaluation, mental health assessment

## Abstract

**Objective:** To analyze the discrepancy between self-rating and professional evaluation of mental health status in coronavirus disease 2019 (COVID-19) cluster cases.

**Method:** A total of 65 COVID-19 cluster cases admitted to Beijing Ditan Hospital Capital Medical University from June 14, 2020 to June 16, 2020 were included in the study. Mental health assessment was completed by self-rating and professional evaluation. The gaps between self-rating and professional evaluation in different demographic characteristics were compared.

**Results:** The results of self-rating were inconsistent with those of professional evaluation. The gap was statistically different among certain demographic subgroups. As for anxiety, the gaps had remarkable statistics differences in subgroups of sex, monthly income, infection way, and anxiety/depression medical history. Similarly, in the terms of depression, the gaps had significant statistic differences in the subgroups of the medical history of anxiety/depression, history of physical disease, employment status and the insurance type, marriage, education (year), residing in Beijing (year), and the monthly income.

**Conclusion:** Compared to the professional evaluation, patients had a higher self-rating, which may be related to some demographic characteristics. It suggests that screening can be conducted in patients with COVID-19 by self-rating first, and then professional evaluation should be carried out in the patients with suspicious or positive results.

## Introduction

Since the coronavirus disease 2019 (COVID-19) caused by severe acute respiratory syndrome coronavirus 2 (SARS-CoV-2) had been reported in December 2019 (Zhu et al., 2020), it has gained wide attention rapidly. SARS-CoV-2 is highly contagious (Fang et al., 2020), and it could develop into a severe even fatal respiratory system disease (Guan et al., 2020; Huang et al., 2020), as well as develop neurological disorders (Wenting et al., 2020; Taquet et al., 2021).

Studies show clear signs of the harmful consequences, both mentally and psychologically, of COVID-19 in the general population (e.g., anxiety and depression; World Health Organization, 2020; de Vroege and van den Broek, 2021).

Being similar to other coronaviruses (Mak et al., 2009), patients with COVID-19 might show mental pressure, anxiety and depression syndromes, psychiatric dysfunction, and other such problems (Li et al., 2020a; Liu et al., 2020; Pappa et al., 2020; Qi et al., 2020; Torales et al., 2020; Zhang et al., 2020), even in the long term after the disease (Taquet et al., 2021). It is possibly because the patients experienced fear of the consequences of severe disease and the contagion (Xiang et al., 2020). Furthermore, strict quarantine and mandatory contact-tracing policy by the health authorities could cause societal rejection, financial loss, discrimination, and stigmatization (Balsamo and Carlucci, 2020; Brooks et al., 2020; Carlucci et al., 2020; Shigemura et al., 2020; Vindegaard and Benros, 2020). Some patients even committed suicide after experiencing anxiety and insomnia in the beginning of the hospitalization. In Italy, since the beginning of the COVID-19 quarantine, domestic homicides, and murder suicides registered to date increased (Balsamo and Carlucci, 2020). But it remains unclear whether the risks are attributable to viral infections *per se* or the host immune response (Troyer et al., 2020).

Therefore, accurate assessment of the mental status of patients is especially important (Epstein et al., 2020), which can avoid wastage of the limited mental health resources, and patients can be supported through less intense ways. On the contrary, it can also reduce the burden of medical workers. Previous studies showed that there were differences between self-rating and professional evaluation results, and the depression rate reported through self-rating scale was significantly higher than those through clinical interview (Lim et al., 2018). For the slightly depression conditions, more depression symptoms can be generated through self-rating (Rush et al., 1987).

Professional evaluation and self-rating are two common clinical scales, and can provide important information for clinical doctors (Möller, 2000). Self-rating, which is economical and practical, is widely used for survey and screening procedures, especially for slight diseases such as anxiety (Maier et al., 1990). The problem is the self-awareness of the patients may not reflect reality. It's hard to complete for some patients lack of reading ability (Hamilton, 1976). While professional evaluation is a comprehensive evaluation to the symptoms of the patients by clinically trained professionals with experience, which is still the golden standard for mental disorder diagnosis (Dunstan et al., 2017).

The self-rating anxiety scale (SAS) and self-rating depression scale (SDS) of Zung are the widely used for anxiety and depression evaluation (Zung, 1965, 1971; Möller, 2000). The Hamilton Anxiety (HAMA) and the Hamilton Depression (HAMD) rating scales are commonly used in evaluating anxiety and depression (Hamilton, 1959, 1960). They are the golden standard in the depression evaluation (Bagby et al., 2004). During the COVID-19 pandemic, it was widely used to investigate the psychological status of the different population (Li et al., 2020b).

Most of the researches on the psychological evaluation of patients with COVID-19 used self-rating scales (Guo et al., 2020), and the application of the professional evaluations is rare. Besides, the rare research has reported the difference between self-rating and professional evaluation in the patients with COVID-19. This study conducted that both self-rating and professional evaluation in COVID-19 cluster cases, and compared the results obtained by these two methods.

## Materials and Methods

### Participants and Data Collection

There were 65 cluster patients with COVID-19 enrolled in this study. All of them were admitted to Beijing Ditan Hospital from June 14, 2020 to 16 June 16, 2020, including 38 male patients, aging from 21 to 65 (42.29 ± 12.344). This is a cross-sectional observational study that is approved by the Ethics Committee of Beijing Ditan Hospital affiliated to Capital Medical University.

The inclusion criteria were: (i) age >18, (ii) diagnosed as COVID-19 (mild-type to normal-type), which was based on the COVID-19 diagnosis protocol (trial version 7) issued by the State Health Committee, and (iii) patient informed consent.

The exclusion criteria were: (i) had a recent history of travel and residence overseas or in other high-risk areas, (ii) had severe psychological trauma recently, (iii) having severe heart, liver, kidney, or nervous system diseases, and (iv) failed to cooperate to complete the self-rating and the professional evaluation.

Demographic data were collected, including sex, age, marital status, educational background, length of residence in Beijing in the years, employment status, monthly income, insurance type, infection way, family member infected or not, smoking history, psychological disease, and other physical disease history.

### Measurement of Psychological Traits and Procedures

This study, through online survey, conducted self-evaluation by using SAS and SDS. The Chinese norm shows that the cut-off value of the SAS standard score is 50. From 50 to 59 points was slightly anxiety, 60 to 69 points were medium anxiety, and above 70 points is heavily anxiety. The Chinese norm shows that the cut-off value of the SDS standard score is 53. From 53 to 62 is slightly depression, 63 to 72 is medium depression, and above 72 is heavily depression.

In addition, the mental health of the patients was assessed by the two trained attending psychiatrists, who passed the consistency evaluation, through with HAMA and HAMD.

Hamilton Anxiety rating scale items were concluded into two dimensions: (i) mental anxiety and (ii) somatic anxiety. HAMD items were concluded into seven dimensions: (i) Anxiety/somatization: consisting of mental anxiety, physical anxiety, digestive symptoms, hypochondria, and insight, (ii) Weight: weight loss item, (iii) Cognitive disorder: consisting of self-guilt, suicidal, intense, depersonalization and derealization, paranoid symptoms, and obsessive compulsive symptoms, (iv) Diurnal variation: only this item, (v) Retardation: consisting of the depressive emotions, work and hobby, retardation, and sex symptoms, (vi) Sleeping disorder: consisting of the difficulty falling into sleep, sleep light and early wakeup, (vii) Desperation: consisting of the sense of diminished ability, desperation and sense of inferiority. HAMA ≥7, anxiety symptoms exist and HAMD ≥8, depression symptoms exist.

### Statistical Analysis

The SPSS 17.0 software was used to analyze the data. Quantitative data with normal distribution were expressed using the format mean ± SD ($\bar{x}$ ± s). The *t*-test for the two isolated samples was used to make comparison between the two groups. One-way ANOVA was used to compare multiple groups. Mann–Whitney *U*-test was used to make comparison between two quantitative data groups with non-normal distribution. The Kruskal–Wallis test was used to make comparison among multiple isolated samples with non-normal distribution, and *p* < 0.05 was considered statistically significant.

We calculate the gaps between the self-rating scale and professional evaluation in the same dimension. However, the self-rating scale and professional evaluation have completely different measurement and the scoring system. So, 0–1 normalization process is used to standardize the scores in each dimension, which makes the dimension scores comparable between the self-rating scale and the professional evaluation. The 0–1 normalization process of the *i*th question for the *j*th patient is calculated as follow:

$$\begin{array}{l} Standard_{i}=\frac{score_{ij}-min\left(score_{i}\right)}{max\left(score_{i}\right)-min\left(score_{i}\right)},i=1,\ldots 1\;24,\\ \;j=1,\ldots 1\;58 \end{array}$$

*Standard*_*i*_ is the standardized scores for the *i*th question, *score*_*ij*_ is the initial score of the *i*th question for the *j*th patient in the HAMD scale. Max(*score*_*i*_) *and min*(*score*_*i*_) are the theoretical maximum and minimum values of the scores in the *i*th question. Take the HAMD scores as an example, max(*score*_*i*_)−*mi*n(*score*_*i*_) = 4 − 0 = 4. We conduct this process for all the questions in the HAMD scale, and then calculate the average standardize scores in each dimension. By this way, we get the standard scores in seven dimensions in the HAMD scale. After the 0–1 normalization, the maximum standard score becomes 1 and the standard minimum score becomes 0. The exactly same process is also conducted in the SDS scale and their anxiety evaluation counterparts. Although the self-rating scale and the professional evaluation have different measurement in both the anxiety and depression evaluation, the 0–1 normalization process makes it possible to make a compare of one particular dimension between the self-rating scale and the professional evaluation. The gap between self-rating and professional evaluation within one particular dimension is calculated by the standard professional evaluation score minus standard self-rating score.

All the data collected by the scales were stored in the database. Then, the calculation functions in the database could help to finish the 0–1 normalization process, and the statistical analysis was running based on these data. So, the methods allow for the replication studies.

## Results

A total of 65 patients participated in this study, and 58 patients had finished both the self-rating and the professional evaluation.

**Figure 1:**
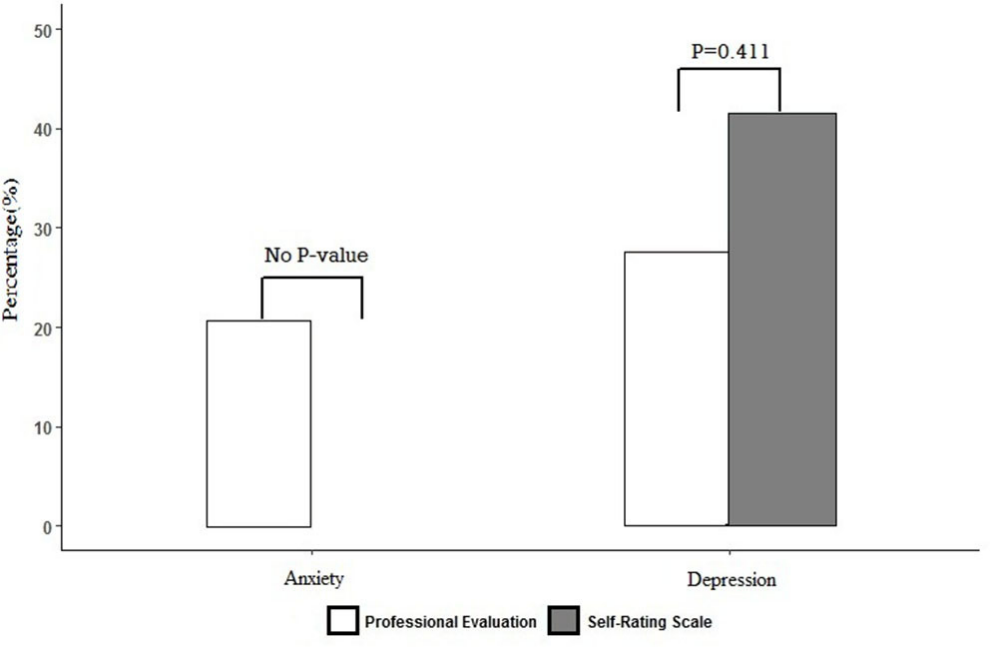
Result of SAS vs. HAMA and SDS vs. HAMD.

The self-rating is inconsistent with the professional evaluation as Figure 1 shows Kappa Consistence.

### The Anxiety Gap Values Between Self-Rating and Professional Evaluation

**Figure 2:**
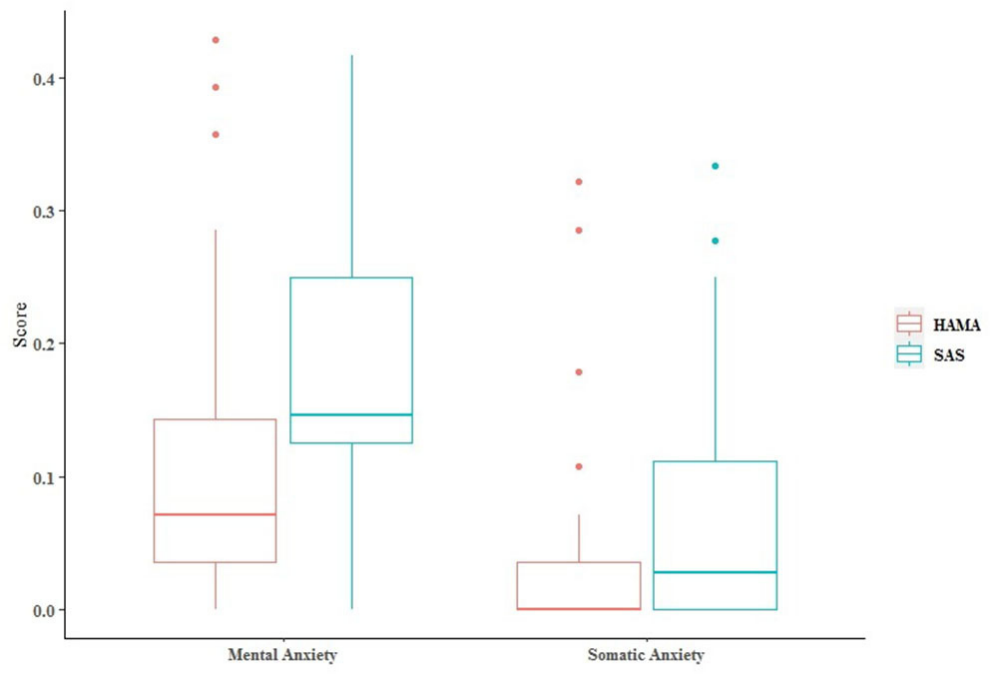
The SAS was divided and matched by referring to the two dimensions of somatic anxiety and mental anxiety in HAMA, and the scores of the same dimension were compared.

The SAS score was (31.79 ± 4.742), with anxiety symptoms 0 case. The HAMA score was (4.16 ± 4.043), 12 cases (20.7%) had anxiety symptoms. The scores of two dimensions of SAS were both higher than HAMA (Figure 2), and the differences were statistically significant (*p* = 0.001, *p* = 0.018).

The value of *p* < 0.05 indicates that the factors are the reason for the significant difference in the gaps, and that may be the reason for the difference between the results of SAS and HAMA (Table 1). Among/between the subgroups of sex, monthly, and anxiety/depression medical history, the mental anxiety gap had significant difference. Between the subgroups of the infection way, the somatic anxiety gap had significant difference.

**Table 1:** Gaps of SAS and HAMA scores among various demographic characteristics.

**Characteristics**	**All patients *N* = 58 (%)**	**Mental anxiety gap^**#**^**	***F/t***	***p*-value**	**Somatic anxiety gap^**#**^**	***F/t***	***p*-value**
Sex			−2.489	0.016^a^		0.059	0.953^a^
Male	32 (55.2)	(−0.11 ± 0.136)			(−0.03 ± 0.072)		
Female	26 (44.8)	(−0.02 ± 0.149)			(−0.03 ± 0.114)		
Age			1.376	0.261^b^		0.426	0.655^b^
<40	29 (50.0)	(−0.05 ± 0.131)			(−0.03 ± 0.078)		
40–60	24 (41.4)	(−0.10 ± 0.146)			(−0.04 ± 0.112)		
>60	5 (5.8)	(0.00 ± 0.235)			(0.00 ± 0.062)		
Marriage			0.521	0.597^b^		0.095	0.910^b^
Not married	4 (6.9)	(−0.08 ± 0.061)			(−0.03 ± 0.075)		
Married	51 (87.9)	(−0.07 ± 0.154)			(−0.03 ± 0.096)		
Divorced/widowed	3 (5.1)	(0.02 ± 0.117)			(0.00 ± 0.046)		
Education (year)			0.380	0.686^b^		0.514	0.601^b^
<6	11 (19.0)	(−0.04 ± 0.095)			(0.00 ± 0.143)		
6~9	29 (50.0)	(−0.08 ± 0.160)			(−0.03 ± 0.081)		
>9	18 (41)	(−0.06 ± 0.157)			(−0.04 ± 0.072)		
Residing in Beijing (year)			1.564	0.218^b^		0.285	0.753^b^
<3	15 (25.9)	(−0.06 ± 0.145)			(−0.01 ± 0.080)		
3~10	16 (27.6)	(−0.02 ± 0.102)			(−0.03 ± 0.106)		
>10	27 (46.5)	(−0.10 ± 0.168)			(−0.04 ± 0.093)		
Employment status			−1.396	0.168^a^		0.381	0.705^a^
No	29 (50%)	(−0.04 ± 0.142)			(−0.03 ± 0.105)		
Yes	29 (50%)	(−0.09 ± 0.152)			(−0.02 ± 0.079)		
Monthly (RMB)			3.175	0.031^b^		0.494	0.688^b^
<2,000	8 (13.8)	(0.01 ± 0.128)			(−0.06 ± 0.080)		
2,000–5,000	32 (55.2)	(−0.04 ± 0.125)			(−0.02 ± 0.091)		
5,000–10,000	14 (24.1)	(−0.13 ± 0.172)			(−0.03 ± 0.101)		
10,000–20,000	4 (6.9)	(−0.20 ± 0.153)			(−0.05 ± 0.111)		
Insurance type			0.690	0.506^b^		0.789	0.459^b^
Social	24 (41.4)	(−0.07 ± 0.158)			(−0.03 ± 0.083)		
Rural	27 (46.6)	(−0.08 ± 0.142)			(−0.04 ± 0.085)		
Other	7 (12.1)	(0.00 ± 0.141)			(0.01 ± 0.146)		
Family member infected			0.072	0.943^a^		−0.249	0.804^a^
Yes	18 (31.0)	(−0.06 ± 0.169)			(−0.03 ± 0.100)		
No	40 (69.0)	(−0.07 ± 0.140)			(−0.03 ± 0.090)		
Physical disease history^**^			0.272	0.791^a^		1.106	0.273^a^
Yes	10 (17.2)	(−0.06 ± 0.133)			(−0.02 ± 0.098)		
No	48 (82.8)	(−0.08 ± 0.214)			(−0.06 ± 0.055)		
Anxiety/depression medical history^*^			−2.150	0.036^a^		0.295	0.769^a^
Yes	4 (6.9)	(−0.08 ± 0.145)			(−0.03 ± 0.095)		
No	54 (93.1)	(0.08 ± 0.122)			(−0.04 ± 0.031)		
Smoking			1.200	0.235^a^		0.663	0.529^a^
Yes	16 (27.6)	(−0.05 ± 0.146)			(−0.02 ± 0.093)		
No	42 (72.4)	(−0.10 ± 0.152)			(−0.04 ± 0.093)		
Infection way			1.966	0.150^b^		3.568	0.035^b^
Working at Xinfadi	26 (44.8)	(−0.10 ± 0.160)			(−0.04 ± 0.098)		
Activity at Xinfadi	15 (25.9)	(0.00 ± 0.144)			(−0.06 ± 0.077)		
Indirect contact	17 (29.3)	(−0.06 ± 0.120)			(0.02 ± 0.081)		

a *t-test*.

b *One-way ANOVA*.

* *Past medical history*.

** *Hypertension, diabetes, Cirrhosis after hepatitis B, post-operative breast cancer*.

# *Gap = the standardized score of HAMA minus the standardized score of SAS in the same dimension*.

### The Depression Gap Values Between Self-Rating and Professional Evaluation

**Figure 3:**
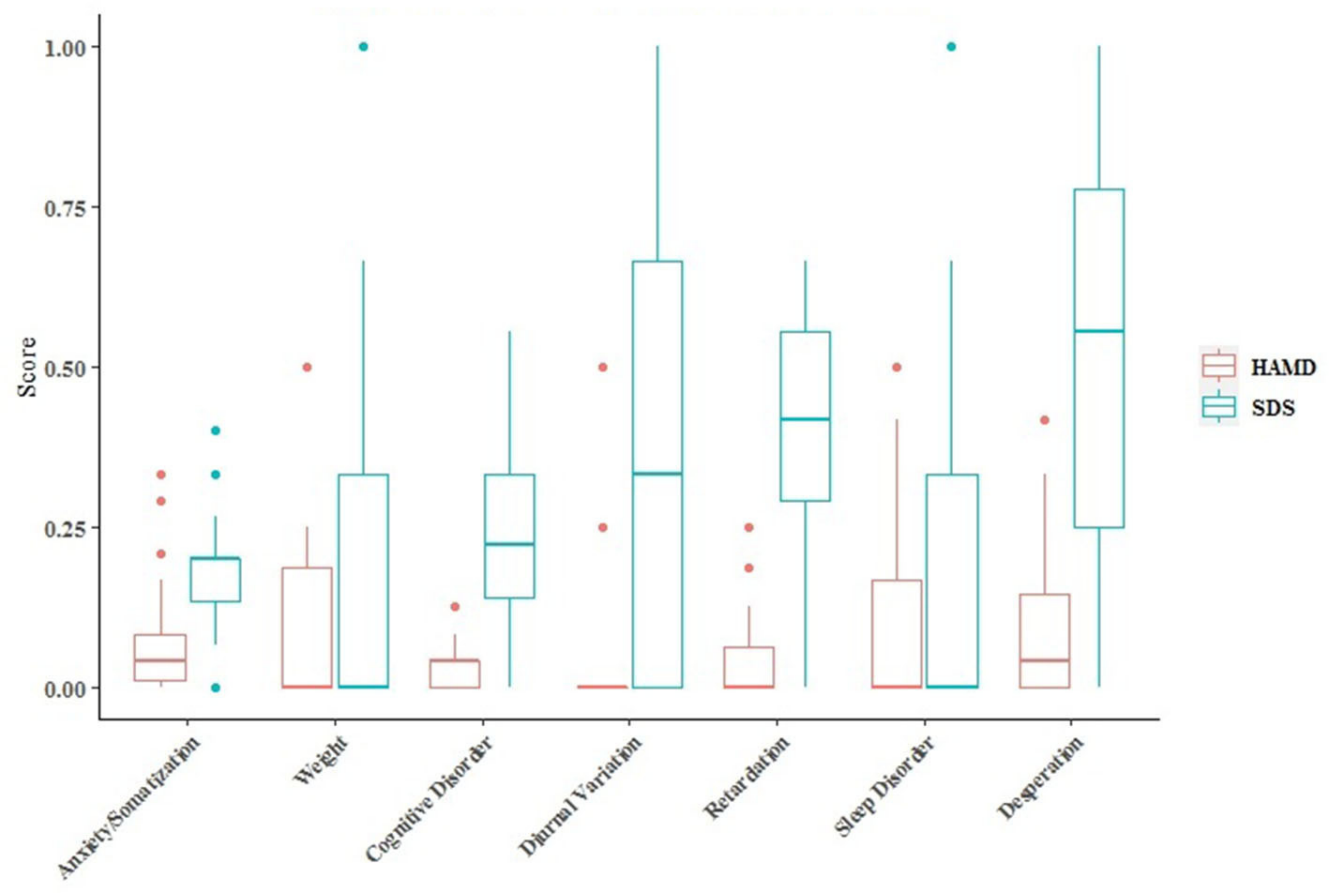
The SDS scale was divided and matched according to the seven dimensions of HAMD, and the scores of the same dimension were compared.

The SDS score was (38.55 ± 8.664), 24 cases (41.4%) had depression symptoms, including 19 cases of mild depression, and 5 cases of moderate depression. The HAMD score was (6.10 ± 4.734), 16 cases (27.6%) had depressive symptoms, all were slightly depressed. The SDS scores on seven dimensions were all higher than HAMD (Figure 3), and the differences were statistically significant, *p* < 0.05.

The value of *p* < 0.05 indicates that the factors are the reason for the significant difference in the gaps, and that may be the reason for the difference between the results of the SDS and HAMD (Table 2).

**Table 2:** Gaps of SDS and HAMD scores among various demographic characteristics.

**Items**	**All patients** ***N* = 58 (%)**	**Anxiety/** **Somatization gap^**#**^**	***p*-value**	**Cognitive gap^**#**^**	***p*-value**	**Retardation gap^**#**^**	***p-*value**	**Desperation gap^**#**^**	***p*-value**	**Weight gap^**#**^**	***p*-value**	**Diurnal variation gap^**#**^**	***p*-value**	**Sleeping gap**	***p*-value**
Sex			0.194^a^		0.942		0.310 ^a^		0.855^a^		0.083^c^		0.441^c^		0.518^c^
Male	32 (55.2)	(−0.12 ± 0.105)		(−0.20 ± 0.168)		(−0.38 ± 0.201)		(−0.45 ± 0.345)		(−0.14 ± 0.316)		(−0.40 ± 0.340)		(−0.10 ± 0.165)	
Female	26 (44.8)	(−0.08 ± 0.112)		(−0.21 ± 0.134)		(−0.32 ± 0.194)		(−0.43 ± 0.387)		(0.00 ± 0.235)		(−0.33 ± 0.332)		(−0.08 ± 0.204)	
Age			0.461^b^		0.904^b^		0.568^b^		0.774^b^		0.134^d^		0.218^d^		0.817^d^
<40	29 (50.0)	(−0.10 ± 0.122)		(−0.20 ± 0.153)		(−0.34 ± 0.218)		(−0.43 ± 0.323)		(−0.02 ± 0.301)		(−0.29 ± 0.307)		(−0.10 ± 0.183)	
40–60	24 (41.4)	(−0.11 ± 0.082)		(−0.21 ± 0.158)		(−0.38 ± 0.180)		(−0.43 ± 0.408)		(−0.16 ± 0.279)		(−0.44 ± 0.336)		(−0.10 ± 0.193)	
>60	5 (5.8)	(−0.04 ± 0.145)		(−0.20 ± 0.155)		(−0.28 ± 0.170)		(−0.56 ± 0.394)		(−0.02 ± 0.207)		(−0.47 ± 0.447)		(−0.02 ± 0.124)	
Marriage			0.739 ^b^		0.358^b^		0.026^b^		0.595^b^		0.889^d^		0.172^d^		0.485^d^
No	4 (6.9)	(−0.08 ± 0.097)		(−0.16 ± 0.121)		(−0.43 ± 0.053)		(−0.50 ± 0.192)		(−0.08 ± 0.167)		(−0.25 ± 0.167)		(0.00 ± 0.000)	
Yes	51 (87.9)	(−0.10 ± 0.110)		(−0.21 ± 0.152)		(−0.37 ± 0.197)		(−0.45 ± 0.378)		(−0.08 ± 0.303)		(−0.40 ± 0.343)		(−0.09 ± 0.179)	
Divorced/Widowed	3 (5.1)	(−0.06 ± 0.129)		(−0.09 ± 0.209)		(−0.07 ± 0.085)		(−0.24 ± 0.140)		(−0.11 ± 0.192)		(−0.03 ± 0.048)		(−0.25 ± 0.300)	
Education (year)			0.191^b^		0.080^b^		0.032^b^		0.047^b^		0.532^d^		0.302^d^		0.754^d^
<6	11 (19.0)	(−0.06 ± 0.096)		(−0.12 ± 0.124)		(−0.28 ± 0.208)		(−0.22 ± 0.375)		(−0.08 ± 0.423)		(−0.27 ± 0.291)		(−0.14 ± 0.205)	
6~9	29 (50.0)	(−0.09 ± 0.103)		(−0.20 ± 0.182)		(−0.32 ± 0.201)		(−0.45 ± 0.402)		(−0.05 ± 0.288)		(−0.43 ± 0.349)		(−0.07 ± 0.168)	
>9	18 (41)	(−0.13 ± 0.121)		(−0.25 ± 0.089)		(−0.45 ± 0.154)		(−0.56 ± 0.207)		(−0.11 ± 0.190)		(−0.32 ± 0.336)		(−0.11 ± 0.193)	
Residing in Beijing (year)			0.458^b^		0.896^b^		0.024^b^		0.135^b^		0.329^d^		0.153^d^		0.848^d^
<3	15 (25.9)	(−0.09 ± 0.101)		(−0.20 ± 0.177)		(−0.32 ± 0.134)		(−0.41 ± 0.319)		(0.01 ± 0.194)		(−0.38 ± 0.305)		(−0.08 ± 0.159)	
3~10	16 (27.6)	(−0.08 ± 0.120)		(−0.19 ± 0.162)		(−0.26 ± 0.225)		(−0.32 ± 0.343)		(−0.08 ± 0.374)		(−0.23 ± 0.287)		(−0.07 ± 0.149)	
>10	27 (46.5)	(−0.12 ± 0.107)		(−0.21 ± 0.137)		(−0.43 ± 0.190)		(−0.54 ± 0.378)		(−0.13 ± 0.274)		(−0.44 ± 0.364)		(−0.11 ± 0.213)	
Employment status			0.444^a^		0.200^a^		0.243^a^		0.060^a^		0.179^c^		0.248^c^		0.014^c^
No	29 (50%)	(−0.09 ± 0.108)		(−0.23 ± 0.135)		(−0.38 ± 0.204)		(−0.53 ± 0.362)		(−0.12 ± 0.332)		(−0.31 ± 0.305)		(−0.13 ± 0.157)	
Yes	29 (50%)	(−0.11 ± 0.111)		(−0.18 ± 0.167)		(−0.32 ± 0.190)		(−0.35 ± 0.343)		(−0.03 ± 0.236)		(−0.42 ± 0.360)		(−0.05 ± 0.200)	
Monthly (RMB) income (RMB)			0.217^b^		0.086^b^		0.163^b^		0.026^b^		0.991^d^		0.020^d^		0.205^d^
<2,000	8 (13.8)	(−0.14 ± 0.106)		(−0.10 ± 0.120)		(−0.26 ± 0.223)		(−0.16 ± 0.358)		(−0.08 ± 0345)		(−0.25 ± 0.295)		(−0.20 ± 0.178)	
2,000–5,000	32 (55.2)	(−0.07 ± 0.108)		(−0.22 ± 0.115)		(−0.35 ± 0.156)		(−0.47 ± 0.328)		(−0.08 ± 0.273)		(−0.33 ± 0.298)		(−0.10 ± 0.192)	
5,000–10,000	14 (24.1)	(−0.12 ± 0.113)		(−0.19 ± 0.156)		(−0.36 ± 0.262)		(−0.45 ± 0.338)		(−0.07 ± 0.336)		(−0.36 ± 0.351)		(−0.04 ± 0.149)	
10,000–20,000	4 (6.9)	(−0.16 ± 0.087)		(−0.31 ± 0.076)		(−0.53 ± 0.072)		(−0.80 ± 0.166)		(−0.08 ± 0.215)		(−0.92 ± 0.167)		(−0.06 ± 0.185)	
Insurance type			0.034^b^		0.593^b^		0.812^b^		0.823^b^		0.492^d^		0.364^d^		0.828^d^
Social	24 (41.4)	(−0.13 ± 0.109)		(−0.21 ± 0.152)		(−0.37 ± 0.205)		(−0.48 ± 0.360)		(−0.02 ± 0.216)		(−0.36 ± 0.345)		(−0.10 ± 0.194)	
Rural	27 (46.6)	(−0.10 ± 0.099)		(−0.20 ± 0.158)		(−0.35 ± 0.199)		(−0.42 ± 0.382)		(−0.12 ± 0.362)		(−0.42 ± 0.341)		(−0.09 ± 0.185)	
Other	7 (12.1)	(0.00 ± 0.110)		(−0.22 ± 0.156)		(−0.31 ± 0.194)		(−0.41 ± 0.321)		(−0.10 ± 0.163)		(−0.20 ± 0.254)		(−0.06 ± 0.162)	
Family member infected			0.097^a^		0.636^a^		0.625^a^		0.720^a^		0.584^c^		0.383^c^		0.623^c^
Yes	18 (31.0)	(−0.13 ± 0.103)		(−0.22 ± 0.175)		(−0.37 ± 0.199)		(−0.47 ± 0.368)		(−0.10 ± 0.318)		(−0.41 ± 0.287)		(−0.10 ± 0.193)	
No	40 (69.0)	(−0.08 ± 0.109)		(−0.20 ± 0.023)		(−0.35 ± 0.200)		(−0.43 ± 0.362)		(−0.07 ± 0.279)		(−0.35 ± 0.357)		(−0.09 ± 0.179)	
Physical disease history^**^			0.125^a^		0.012^a^		0.399^a^		0.591^a^		0.416^c^		0.814^c^		0.322^c^
Yes	10 (17.2)	(−0.09 ± 0.103)		(−0.18 ± 0.145)		(−0.34 ± 0.208)		(−0.43 ± 0.369)		(−0.07 ± 0.301)		(−0.37 ± 0.335)		(−0.09 ± 0.185)	
No	48 (82.8)	(−0.15 ± 0.130)		(−0.32 ± 0.141)		(−0.40 ± 0.139)		(−0.50 ± 0.331)		(−0.13 ± 0.233)		(−0.35 ± 0.355)		(−0.12 ± 0.172)	
Anxiety/depression medical history^*^			0.373^a^		0.156^a^		0.627^a^		0.749^a^		0.647^c^		0.191^c^		0.038^c^
Yes	4 (6.9)	(−0.10 ± 0.107)		(−0.20 ± 0.153)		(−0.36 ± 0.202)		(−0.44 ± 0.369)		(−0.08 ± 0.294)		(−0.38 ± 0.331)		(−0.08 ± 0.177)	
No	54 (93.1)	(−0.04 ± 0.130)		(−0.31 ± 0.125)		(−0.31 ± 0.130)		(−0.50 ± 0.256)		(−0.02 ± 0.239)		(−0.15 ± 0.362)		(−0.27 ± 0.185)	
Smoking			0.514^a^		0.159^a^		0.570^a^		0.467^a^		0.353^c^		0.226^c^		0.283^c^
Yes	16 (27.6)	(−0.09 ± 0.115)		(−0.19 ± 0.144)		(−0.34 ± 0.194)		(−0.42 ± 0.379)		(−0.07 ± 0.303)		(−0.34 ± 0.345)		(−0.08 ± 0.183)	
No	42 (72.4)	(−0.11 ± 0.092)		(−0.25 ± 0.171)		(−0.38 ± 0.213)		(−0.50 ± 0.315)		(−0.10 ± 0.275)		(−0.38 ± 0.213)		(−0.13 ± 0.180)	
Infection way			0.714		0.651^b^		0.600^b^		0.611^b^		0.910^d^		0.106^d^		0.056^d^
Working at Xinfadi	26 (44.8)	(−0.09 ± 0.100)		(−0.22 ± 0.161)		(−0.38 ± 0.207)		(−0.49 ± 0.414)		(−0.07 ± 0.286)		(−0.46 ± 0.299)		(−0.04 ± 0.175)	
Activity at Xinfadi	15 (25.9)	(−0.10 ± 0.129)		(−0.18 ± 0.141)		(−0.33 ± 0.215)		(−0.41 ± 0.332)		(−0.05 ± 0.271)		(−0.26 ± 0.389)		(−0.17 ± 0.190)	
Indirect contact	17 (29.3)	(−0.12 ± 0.108)		(−0.20 ± 0.154)		(−0.33 ± 0.172)		(−0.40 ± 0.306)		(−0.11 ± 0.332)		(−0.32 ± 0.318)		(−0.10 ± 0.168)	

a *t-test*.

b *One-way ANOVA*.

c *Mann–Whitney U-test*.

d *Kruskal–Wallis test*.

* *The difference between groups was statistically significant, p <0.05*.

** *Hypertension, diabetes, Cirrhosis after hepatitis B, post-operative breast cancer*.

# *Gap = the standardized score of HAMD minus the standardized score of SDS in the same dimension*.

Among the subgroups of the insurance type, the anxiety/somatization gap had significant difference. Between the subgroups of the history of physical disease, the cognitive gap had significant difference. Among/between the subgroups of marriage, education (year), and residing in Beijing (year), the retardation gap had significant difference. Among the subgroups of education (year) and monthly, the desperation gap had significant difference. Among the subgroups of monthly, the diurnal variation gap had significant difference. Between the subgroups of the employment status and anxiety/depression medical history, the sleeping gap had significant difference.

## Discussion

By introducing the concept of gap, this study discussed the discrepancy between self-rating and professional evaluation in the COVID-19 cluster cases for the first time. It showed that SAS and SDS scores in the same dimension were significantly higher than the HAMA and HAMD, indicating that there were significant differences between the self-rating and the professional evaluation. This was consistent with the previous reports (Rush et al., 1987; Enns et al., 2014; Krebber et al., 2014; Lim et al., 2018).

Moreover, the male subgroup and the subgroup with higher income had higher self-rating scores in the mental anxiety dimension, which might be due to the fact that they assume more family and social responsibilities, so that they were more likely to worry about the impact of illness on themselves, family life, and the career. Notably, as compared with the indirect contact with Xinfadi, where COVID-19 broke out, the patients with direct contact had higher self-rating of the somatic anxiety, who might be more in need of the attention of doctors (Troyer et al., 2020), which is possibly because the cluster disease increased their feelings of fear and guilt to some extent.

In terms of depression, patients who were non-insured were more likely to have higher self-rating of anxiety/somatic status, which might be due to their fear of bearing high-medical costs. The patients without a history of physical disease had a higher self-rating of cognitive, which might be due to the greater psychological burden of the hospitalization because they had not been hit by the disease. Compared with the patients who were divorced and widowed, the patients who were unmarried and married had higher self-rating of retardation, which might be due to their concern about the impact on their relationship or the family life. The patients with higher education level had higher self-rating of cognitive and desperation status. It was probably because the patients with better education background had better reading ability and could complete the questionnaire accurately, and also knew more about COVID-19 and understood the risk of novel coronavirus infection, which increased their depression mood. The patients with the higher income made a higher self-rating of desperation and diurnal variation, which might be caused by the fear of the disease affecting career and work, and the inability to maintain the current income level. It seemed incomprehensible that the patients without a history of depression tend to have a higher self-rating of sleeping disorder. The patients with a history of depression were able to make a more objective self-rating when they developed insomnia symptoms after being infected with the SARS-CoV-2, which was probably because they had severe insomnia before, and in particular, they had filled the similar rating scales often ever before.

Those differences may be associated with the personality and demography of the patients, which is consistent with the previous studies (Enns et al., 2014). So, when planning to assess the mental health of the patients with COVID-19 by self-rating, it is necessary to pay attention to the age, gender, work status, monthly, education level, employment status, medical history, and infection way of the patients. There may be an unobjective evaluation. For instance, the scores of the two dimensions of anxiety self-rating were both higher than those of the professional evaluation, but the patients with anxiety were not found according to the anxiety self-rating standard, which was not in accordance with the actual situation. We supposed that the SAS diagnostic threshold may need to be adjusted or improved the self-rating questionnaire when used.

Therefore, in the clinical practice, we suggest that screening can be conducted by self-rating first, then the professional evaluation should be carried for the patients with suspicious or positive results, so as to reduce unnecessary contact with the patients and the workload of the medical workers (de Vroege and van den Broek, 2021).

The limitations are the limited number of cases included in the study and the lack of a control group. Fortunately, the included cases were cluster cases, all from Xinfadi where COVID-19 broke, with relatively consistent background and mild condition. As a result those cases are highly homogeneous. It, to some extent, proves something. However, whether self-rating can accurately and effectively assess mental status of the patients and the reasons for the difference between the self-rating and professional evaluation will likely require the closer examination.

## Data Availability Statement

The original contributions presented in the study are included in the article/supplementary material, further inquiries can be directed to the corresponding author/s.

## Ethics Statement

The studies involving human participants were reviewed and approved by the Ethics Committee of Beijing Ditan Hospital affiliated to Capital Medical University. The patients/participants provided their written informed consent to participate in this study.

## Author Contributions

RS developed the ideas in the paper and provided feedback on several drafts of the paper. LL collected the data of demographic and self-rating, and wrote the paper. XW and JY evaluated the patients by HAMA and HAMD. YH conducted statistical analysis of the data in this study. DY, XD, GF, BH, CJ, JL, YL, and YW introduced the study to the patients and obtain their consent. All authors contributed to the article and approved the submitted version.

## Conflict of Interest

The authors declare that the research was conducted in the absence of any commercial or financial relationships that could be construed as a potential conflict of interest.

## Publisher's Note

All claims expressed in this article are solely those of the authors and do not necessarily represent those of their affiliated organizations, or those of the publisher, the editors and the reviewers. Any product that may be evaluated in this article, or claim that may be made by its manufacturer, is not guaranteed or endorsed by the publisher.
